# Molecular phylogenies of the plague microbe *Yersinia pestis*: an environmental assessment

**DOI:** 10.3934/microbiol.2023036

**Published:** 2023-10-16

**Authors:** Victor V. Suntsov

**Affiliations:** A.N. Severtsov Institute of Ecology and Evolution of the Russian Academy of Sciences, Moscow 119071, Russia

**Keywords:** Yersinia *pseudotuberculosis*, *Yersinia pestis*, phylogeny, ecological traits, molecular markers

## Abstract

Two approaches are applied to studies of the phylogeny of the plague microbe *Yersinia pestis*, i.e., the reconstruction of its history: Molecular genetic (MG) and ecological (ECO). The MG approach dominates. Phylogenies created with MG and ECO methods are not congruent. MG conclusions contradict the known facts and patterns of ecology, biogeography, paleontology, etc. We discuss some obvious contradictions and inconsistencies and suggest that real phylogenies of the plague microbe can be constructed only on the basis of the integration of MG and ECO approaches.

## 1. Introduction

In the second half of the 20th century, the theory of natural foci of plague (sylvatic plague) was developed. It attempted to reveal the origin and describe the evolution of the causative agent of infection, the microbe *Yersinia pestis*. However, the knowledge accumulated by that time was extremely limited. The methods of phylogenetic constructions used in earlier empirical-intuitive Haeckelian phylogenetics are fairly primitive from the point of view of modern phylogenetics. Three basic requirements for phylogenic reconstructions were lacking: the analyzed traits were not defined, and methods of analysis and evolutionary models for these traits were not developed.

Rare attempts to interpret the history of the causative agent of plague were based on general theoretical and empirical knowledge accumulated by medical and biological sciences [1]–[3]. The concept of the pathogen's history was based on obvious facts of natural science: The geographical distribution of plague foci in the world; landscape distribution of foci in arid mountainous areas, steppes, semi-deserts and deserts; stable circulation of the microbe in populations of burrowing rodents with a heavy load of fleas; paleontology and paleogeography of burrowing rodents; sensitivity of the major natural hosts of the microbe to the causative agent of plagues, etc. The commonly accepted idea of the emergence of the plague in ancient times arose from this knowledge. High morphological and biochemical similarity with intestinal microbes of the *Enterobacteriaceae* family, the causative agent of pseudotuberculosis (*Y. pseudotuberculosis*) in particular, favored the concept of development of the plague microbe from a pseudotuberculosis-like ancestor living in the digestive tract of rodents or their parasitic fleas. According to the sylvatic plague concept, the plague microbe developed in the course of the long-term co-evolution with rodent hosts and flea vectors in the Oligocene–Pliocene, 30–5 million years ago. The original hosts of the plague microbe were gerbils (Gerbillinae) or marmots (Marmotini) in the Old or the New World. The global expansion of plague was linked to the dispersal of the pathogen by rodent hosts from the center of speciation along the Beringian, Sinai and Panama intercontinental land bridges that regularly arose in geological time and with coevolution and host specialization in populations of new burrowing hosts encountered on expansion routes. The sylvatic plague concept failed to provide an explanation for the mechanistic (primitive) mechanism of the microbe transmission by fleas since fleas are effective in transmitting the pathogen due to the formation of a bacterial plug (a kind of ‘bone in the throat’) in the anterior parts of the digestive tract that blocks the entrance to proventriculus.

The development of genomics and molecular genetic (MG) methods at the turn of the 21st century and their introduction into plague infectology led to two cornerstone discoveries that made significant changes in the provisions of the theory of sylvatic plague. First, contrary to the ideas about the antiquity of the plague, molecular methods showed the evolutionary youth of its causative agent. The molecular clock technique indicated that the plague microbe had emerged no earlier than 30 thousand years ago, i.e., at the boundary between the Pleistocene and the Holocene or in the Holocene [4],[5]. It became clear that speciation occurred in an almost current biogeocenotic environment, when present species of the pathogen hosts and vectors, as well as arid landscapes, already existed within the geographical boundaries of current natural foci of plague. Notably, any speciation process takes place following changes in the habitat of the ancestor species or its introduction into a new environment, i.e., into a new ecological niche. What changes in the habitat of the population (clone) of enteropathogenic ancestor could have led to the recent, almost current emergence of the population of the ‘blood’ parasite, the causative agent of the plague? Where and under what circumstances did the process of transition of the ancestral population from the digestive tract to the lymphomyeloid complex of rodents take place? The answers to these questions are the prerogative of ecology *sensu lato*.

Second, genetic methods, the study of O-antigen, revealed the indisputable direct ancestor of the plague microbe. It turned out to be a psychrophilic pathogen of Far Eastern scarlet-like fever (FESLF)–a pseudotuberculous microbe of the 1st serotype *Y. pseudotuberculosos* 0:1b [6],[7]. This psychrophilic saprozoonotic intestinal pathogen is common in populations of a wide range of invertebrates and vertebrates in cold regions of Asia [8],[9]. Outside the host, it multiplies intensively in organic substrates (excrements, animal corpses) in cold conditions. These facts favored the assumption that the recent speciation of the plague microbe from a clone of the FESLF pathogen occurred in the historical time in the cold regions of Central Asia, where the FESLF pathogen is common and where the ‘cold’ natural foci of plague are located. Many environmental facts indicate that Mongolia with its ultracontinental climate could be the place of speciation of the plague microbe [10]. The maximum Sartan cooling in North and Central Asia, Siberia and the Far East 22–15 thousand years ago is known to be a crucial event at the turn of the Holocene. This cooling caused deep soil freezing in Central Asia, noticeable biocenotic perturbations, the emergence of new intraspecific forms and speciation and the displacement of animal and plant habitats. Environmental reasons to believe that the Sartan cooling was the main abiotic cause of the plague phenomenon are pretty convincing [11].

These two MG discoveries logically linked numerous facts obtained from molecular genetics, ecology, biogeography, biogeocenology, epizootology, paleobiology, paleoclimatology, microbiology and other sciences. It allowed development of an ecological (*sensu lato*) (ECO) scenario of the emergence and Asian expansion of a new highly pathogenic species *Y. pestis*, which is radically different from the MG scenarios [11],[12]. The comparison of MG and ECO scenarios along with the analysis of the reasons for their incongruence is a real way to get an idea of the origin of the plague.

## 2. MG-phylogenies of *Y. pestis*

**Figure 1.**
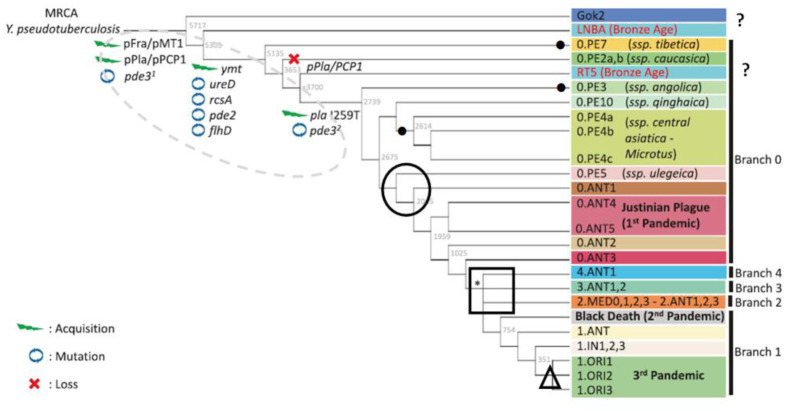
MG-phylogenetic cladogram *Yersinia pestis* [15]. Environmental discrepancies are indicated by icons (see explanations in the text). ●–dubious subspecies; ○–the transformation of the ‘vole’ subspecies (0.PE) into the ‘marmot’ subspecies (0.ANT) on the eve of the 1st pandemic; □–polytomy in Mongolian marmot (*Marmota sibirica*) populations on the eve of the 2nd pandemic; ∆–development of ‘rat’ genovariants (1.ORI) from ‘marmot’ genovariants (1.IN) in Yunnan; ?–extinction of ‘archaeological subspecies' (not justified ecologically [18]). The dotted oval outlines genetic ‘speciation’ changes in the genome of the pseudotuberculous ancestor of the plague microbe.

The phylogenetics of the plague microbe is presently dominated by the MG approach. The most popular phylogenetic schemes are more often presented on dendrograms or cladograms [4],[5],[13]–[17]. The methodology most popular in recent years is based on the analysis of SNP traits, the theory of neutral evolution as the theoretical background of phylogenetic reconstructions of *Y. pestis*, and the use of such statistical methods as estimation of maximum likelihood and Bayesian Information Criterion. Furthermore, the MG methodology for reconstructing the history of the plague microbe borrows techniques developed to study the history (phylogeny) of species, genera, and higher taxa with usually unknown founding species of phylogenetic groups. In addition, new MG methods and procedures are not perfect and the results obtained with the use of them may lead to contradictory and hypothetical conclusions. Therefore, the adequacy of phylogenetic hypotheses put forward in studies that used the MG approach requires verification by other available methods. We discuss here the popular MG-phylogenetic scheme proposed by C. Demeure et al. [15] and evaluate its compliance with environmental facts (Figure 1).

### 2.1. Molecular scenario versus genetic scenario

The inherent inconsistency of the MG approach in the construction of phylogenies is especially noteworthy. Molecular and genetic arguments lack logical unity. From the point of view of genetic transformations, speciation of *Y. pestis* is believed to be a saltation process of ‘horizontal’ introduction of specific virulence plasmids pFra and pPst into the cell of the pseudotuberculous ancestor from the environment or other microorganisms, as well as one-act deletions and inactivation of genes that have lost their functions in a new environment [19],[20]). Genetic transformations are regarded as a result of rapid (‘in eye blink’ [21]) adaptatiogenesis. Moreover, molecular transformations are considered in terms of the theory of neutral evolution as a gradual process of accumulation of neutral minor mutations subject to random drift [4],[5]. Topology of the phylogenetic tree is based only on the statistical analysis of neutral nucleotide substitutions. The presence of genetic adaptive transformations, which actually induced the emergence of the species *Y. pestis* and the entire intraspecific diversity, is only stated (outlined by the dotted oval in Figure 1). In our opinion, disregard for adaptatiogenesis in molecular phylogenetic reconstructions of the plague microbe is the reason for the lack of congruence between MG and ECO phylogenies.

### 2.2. Two-stage Asian expansion of the plague pathogen

MG methods have shown that the most ancient forms of the plague microbe are ‘vole’ subspecies (biovar Pestoides, cluster 0.PE). The nucleotide structure of the analyzed markers reveals their closest proximity to the pseudotuberculous ancestor. The causative agents of the ‘vole’ plague exhibit selective virulence; they are weakly virulent for marmots, ground squirrels, gerbils and humans. The MG approach supposes that after the emergence of *Y. pestis* (Pestoides) in Asia [13] and/or in Africa [22], the range of ‘vole’ foci of 0.PE genovariants formed (Figure 1). Only thousands of years later, on the eve of the 1st pandemic (‘Plague of Justinian’, 541), the causative agent of one of the ‘vole’ subspecies became highly virulent in the populations of the Altai marmot (*Marmota baibacina centralis*) in the Tien Shan and spread widely in Asia in the second wave. The current area of primary natural foci in the populations of many common burrowing rodents was established. Such a two-wave formation of the zone of natural plague foci in Asia has no ecological, biogeographical and epizootological explanations [9].

### 2.3. Transformation of the ‘vole’ subspecies into the ‘marmot’ subspecies on the eve of the 1st pandemic

The pika subspecies 0.PE5 (*Y. pestis ulegeica*) is believed to be the ‘youngest’ among the ‘vole’ subspecies. In MG phylogenies, it became the precursor of all highly virulent subspecies/genovariants [15]–[17] (Figure 1). 0.PE5 circulates in populations of the Mongolian pika (*Ochotona pallasi pricei*) in the Mongolian and Gobi Altai. The ‘marmot’ subspecies derived (allegedly) from it make up the 0.ANT cluster and are found in the Tien Shan and Pamir (Kyrgyzstan, Tajikistan and western Xinjiang, China). According to the ‘molecular’ logic, on the eve of the 1st pandemic the ‘pika’ subspecies 0.PE5 penetrated from Mongolia to the Tien Shan, where it took hold in the populations of the Altai marmot. Later, it returned to Mongolia as 0.ANT1 genovariant and spread in three geographical populations of the Mongolian marmot (*M. sibirica* subsp. *sibirica*, *caliginosus* and *ssp*.), transforming into genovariants 2.ANT3, 3.ANT2 and 4.ANT1. Such a reverse of the plague causative agent in the Mongol-Tien Shan region is inconceivable from the ecological and biogeographical point of view.

### 2.4. Dubious subspecies

Methods of molecular diagnostics have been used to specify the intraspecific diversity of the plague microbe. First, this applies to taxonomic units–subspecies, which represent an ecogeographic category: A subspecies has a range formed by structured populations that have a certain effective size. More than 30 genovariants (=subspecies) characteristic of natural, anthropogenic and secondary natural plague foci have been described [13],[14]. However, ‘vole’ subspecies 0.PE7 (*Y. pestis tibetica*), 0.PE3 (*Y. pestis angolica*) and 0.PE4 (*Y. pestis central-asiatica*) defined by molecular methods do not meet the criteria of the subspecies.

The subspecies 0.PE7 is believed to be the most ancient [13],[15]. In Qinghai Plateau, eastern Tibet four strains of this subspecies have been identified. Two strains were isolated there from the Siberian jerboa (*Allactaga sibirica*), the other two from plague-stricken humans. However, natural foci of plague with the Siberian jerboa as a main host of the microbe are known nowhere in the world. Human infection from jerboas is unlikely. The most probable source of human infection was the Himalayan marmot (*M. himalayana*), which is abundant on the Qinghai Plateau. These rodents are the main hosts of plague microbe in many natural foci in Tibet and the Himalayas, where marmot genovariants/subspecies 1.IN1 and 1.IN2 dominate.

The subspecies 0.PE3 is known from a single strain isolated from a sick person in Angola and long stored in a bacterium collection in the United States [22]. This strain should be qualified as an individual genotype of unknown origin rather than a subspecies.

A composite subspecies 0.PE4 (*Y. pestis central-asiatica*) [14],[17] is comprised of genovariants circulating in populations of the Carruther's vole (*Microtus carruthersi*) on the Hisar Range, the Silver vole (*Alticola argentatus*) on the Talas Range, the Mongolian pika (*Ochotona pallasi pricei*) in the Altai Mountains and the Brandt's vole (*Lasiopodomys brandti*) in Northeast China. Integration of geographically separated populations of the plague microbe which circulate in foci with the major hosts belonging to different genera, families and even orders (Rodentia and Lagomorpha) of burrowing animals into a single subspecies solely on the basis of the nucleotide similarity of the selected molecular markers seems to be incorrect from the ecological point of view. Any subspecies is characterized by the direct relationship of individuals, the unity of the range and the consistency of ecological traits. From the perspective of ecology, the following four separate subspecies are more legitimate: 0.PE4h (*Y. pestis hissarica*), 0.PE4t (*Y. pestis talassica*), 0.PE4a (*Y. pestis altaica*) and 0.PE4x (*Y. pestis xilingolensis* = 0.PE4m, *Y. pestis microtus*). These subspecies are not directly related, as they form a paraphyletic group of subspecies that emerged on separate occasions, in different geographical areas, and originated from different ancestral populations [11]–[13].

### 2.5. Polytomy of Y. pestis in Mongolian marmot populations on the eve of the 2nd pandemic

All known MG phylogenetic trees of *Y. pestis* show the alleged polytomy (N07, ‘Big Bang’) on the eve of the second pandemic (‘Black Death’, 1346). The polytomy is formed by four phylogenetic branches 1, 2, 3 and 4 (Figure 1), which include subspecies/genovariants of the plague microbe 2.ANT3, 3.ANT2 and 4.ANT1. These subspecies/genovariants occur in plague foci in three geographical populations of the Mongolian marmot semisympatric to the Mongolian pika populations. In this case, it remains unclear why the ‘vole’ subspecies 0.PE5 characteristic of natural foci in the Mongolian and Gobi Altai with abundant Mongolian marmots (*Marmota sibirica*) first formed marmot subspecies/genovariants in the populations of the Altai marmot in the Tien Shan and only much later returned to Mongolia and penetrated into the settlements of the Mongolian marmot. MG approach fails to give a satisfactory explanation of the polytomy that arose in the Middle Ages, whereas the ecological scenario provides a credible explanation, which is worthy of discussion and further research [11],[12].

### 2.6. Paraphyly of the subspecies of the ‘branch’ 0.PE

The ‘vole’ subspecies of the plague pathogen, united in the 0.PE cluster, are believed to be the most ancient and placed closer to the root of the *Y. pestis* phylogenetic tree. The members of the 0.PE cluster are sometimes considered a paraphyletic group, though all genovariants 0.PE are combined in one subspecies *Y. pestis microtus* (*microti*) [23],[24]. It should be noted here that subspecies are defined as monophyletic groups of directly related forms (organisms). The members of paraphyletic groups are not directly related and cannot represent a single subspecies. Thus, the subspecies *Y. pestis microtus* and a separate branch of 0.PE do not exist. The well-known study [13] also assumes that all ‘vole’ subspecies originated from different geographical areas, developed in populations of different main hosts and at different times. Therefore, they are not directly related and, consequently, do not form a monophyletic group 0.PE. Homoplasy development of nucleotide characters indicating the similarity (but not kinship) of all ‘vole’ subspecies is obvious [25]. Homoplasy features should be excluded from phylogenetic reconstructions.

### 2.7. Formation of ‘rat’ genovariants 1.ORI in Yunnan

Genovariants of the phylogenetic branch 1.ORI are responsible for the 3rd pandemic that started in Southeast China in the second half of the 19th century. The MG approach assumes that this branch was formed no earlier than 300–400 years ago from the causative agent of the 1.IN branch circulating in the populations of the Himalayan marmot in southern and eastern Tibet (Figure 1) [15],[16]. The main host of 1.ORI genovariants in Yunnan foci is the Buff-Breasted rat (*Rattus flavipectus*). However, this inhabitant of humid subtropics hosts no specific fleas. In agricultural lands and in human settlements, this rodent is parasitized by *Xenopsylla cheopis* (cosmopolitan flea of African origin) that was brought by domestic rats to southern China no earlier than the mid-19th century. Thus, environmental facts favor the absence of natural rat foci of plague in Yunnan. Ecological data indicate that the 1.ORI branch developed in the early or middle Holocene on the Indian subcontinent in populations of the Indian gerbil (*Tatera indica*) rather than in the last centuries in Yunnan in populations of wild or domestic rats [11]. The plague must be brought to Yunnan by domestic rats from India via Myanmar prior to the onset of the 3rd pandemic in the mid-19th century.

### 2.8. Extinction of ‘archaeological subspecies'

In Europe, fragments of ‘archaeological’ DNA were found in the remains of humans who died from plague during the first and second pandemics. With regard to the nucleotide structure of some markers, these fragments are close to homologous fragments of the pseudotuberculous microbe. Therefore, the ‘archaeological’ taxa/subspecies Gok2, LNBA and the others are placed at the roots of phylogenetic trees of *Y. pestis* [15],[16] (Figure 1). No modern genotypes of the plague microbe with homologous ‘archaeological’ molecular markers have been found, and ‘archaeological’ taxa/subspecies are considered extinct [17]. However, environmental conditions in postglacial Europe are known to have been inappropriate for the persistence of natural plague foci. In the Holocene, no European landscapes were densely populated by burrowing rodents with heavy loads of parasitic fleas [18]. Therefore, there are no ample ecological grounds supporting the extinction of any natural lines in the phylogeny of the plague microbe caused by the extinction of hosts or vectors of the pathogen. The plague foci that have faded away in Europe were undoubtedly synanthropic, with the pathogen introduced from Asia. Epizootics were maintained in populations of domestic rats carrying recent subspecies of the pathogen.

## 3. Ecological scenario

As mentioned above, the MG methodology fails to make provisions for the reconstruction of a realistic history (phylogeny) of the plague pathogen. MG methodologies are intent on revealing probable histories of higher taxa over long time intervals. The parental species and, even more so, the parental populations of the phylogenetic groups are for the most part unknown in classical phylogenetic constructions. The concept of the subjectively chosen external group is applied to the characterization of the root of the phylogenetic tree. A different matter in the study of the history of the causative agent of the plague is the ecological approach targeted at the speciation and clarification of intraspecific relationships. The ancestral form of the plague microbe–the causative agent of FESLF–is known for certain, and there is no need for a subjective choice of a suitable external group. The study of the history of plague is the subject of traditional population ecology and phylogeography operating with such terms and concepts as population, subspecies, intraspecific diversification, range and ecological unity rather than the subject of phylogenetics with its complex statistical stochastic computer technology of historical calculations.

**Figure 2.**
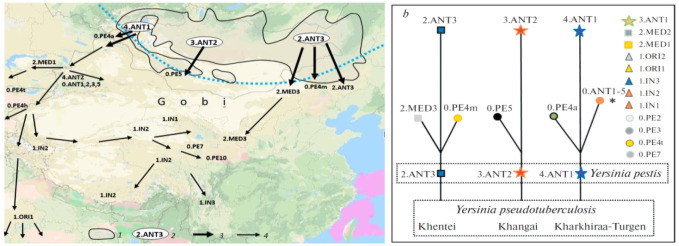
Ecological scenario of speciation and Asian expansion of *Yersinia pestis*. *a* – routes of expansion from areas of speciation [11]. The dotted line is the southern boundary of the permafrost. 1–the range of the Mongolian marmot (*Marmota sibirica*); 2–initial genovariants/subspecies of the plague pathogen in three geographical populations of the Mongolian marmot; 3–routes of distribution of marmot genovariants/subspecies in the populations of burrowing rodents and Mongolian pika sympatric to the Mongolian marmot populations; 4–Asian expansion of the plague microbe with the development of new genovariants/subspecies. *b*–three-rooted ‘ecological’ phylogenetic tree (or phylogenetic grove) [26]. *–diversification and Asian expansion of genovariants.

The causative agent of plague is the sole flea-borne ‘blood’ microbe of the ‘intestinal’ *Enterobacteriaceae* family, which suggests a unique way of its evolution and thus the application of an *ad hoc* evolutionary model for the reconstruction of its history [26],[27]. Two aforementioned MG discoveries related to the evolution of the plague microbe allowed narration of a plausible ecological scenario of its history. The uniqueness of transformation of the population (clone) of the ancestral causative agent of FESLF into the population of the plague microbe, i.e., the speciation, is in infecting the original host of the plague microbe, the Mongolian marmot, not by the traditional alimentary way on pasture, but by the traumatic way during hibernation [26],[27]. The speciation process conformed to the quantum principle. On an evolutionary time scale, it proceeded rapidly, but gradually, with the involvement of such poorly studied phenomena as the oxidative burst of macrophages in the body of heterothermic marmots and stress-induced mutagenesis of the transitional form of *Y. pseudotuberculosis*-*pestis* in populations of hibernating animals [28],[29]. Along with it, parallel speciation processes proceeded in three geographical populations of the Mongolian marmot. Three subspecies/genovariants: 2.ANT3 (Khentei, Barga), 3.ANT2 (Khangai) and 4.ANT1 (Kharkhiraa, Turgen, Mongun-Taiga) demonstrating the ‘Big Bang’ polytomy on MG phylogenetic schemes [10]–[12] emerged (almost) simultaneously (Figure 2). Further spatial expansion of the pathogen in Asia started from the Mongolian centers of speciation and followed independent routes leading to the development of natural foci with specific subspecies/genovariants in the populations of burrowing rodents and the Mongolian pika. This ecological scenario of the origin and evolution of the plague pathogen provides explanations for many contradictions of the MG approach.

## 4. Conclusion

The MG approach includes the following key mechanisms of the evolution of the plague microbe. From the genetic perspective, they are saltation processes: Exogenous horizontal transfer of genetic material (primarily plasmids of virulence pFra and pPst) along with the deletion and inactivation of genes with their functions lost in the new environment. From the molecular perspective, the topology of the phylogenetic tree is derived from the model of stepwise neutral evolution, which provides for the gradual accumulation of neutral mutations and random genetic drift. These two aspects of the MG approach to the problem of the origin of plague are incompatible, conflicting and antagonistic. Therefore, no consistent scenario can be worked out on the basis of the current mainstream MG approach to the evolution and phylogeny of the plague microbe. In addition, evolution is also considered a Darwinian population-genetic process. The history of the species *Y. pestis* is the history of its numerous multi-scale populations living in a variety of rodent-flea environments. Populations of rodents and fleas provide invaluable phylogenetic information about the causative agent of plague, which should be taken into account in historical reconstructions. Therefore, an ECO approach is in demand in solving the problem. Ecological facts indicate that parapatric speciation of the plague microbe occurred no earlier than 30 thousand years ago in Central Asia in three geographical populations of the Mongolian marmot. Three original genovariants/subspecies of the plague microbe 2.ANT3, 3.ANT2 and 4.ANT1 emerged (almost) simultaneously. Further expansion proceeded along independent routes. Such a narrative of the history of the plague microbe allows us to get rid of many contradictions of the MG approach. The history of the ‘young’ causative agent of plague is better reflected in adaptive characteristics, whereas neutral nucleotide characters are more suitable for studying the history of higher taxa over longer geological intervals. The model of neutral evolution can reflect only the main ‘vertical’ axis of the evolution of intestinal bacteria rather than radical innovation and individual adaptations. Furthermore, it is private adaptations that reveal, in detail, the history of the plague microbe.

The proponents of the MG approach speak in terms of the most recent common ancestor (MRCA), phylogenetic branches (branches 0, 1, 2, 3, 4) and evolutionary lines (0.PE, 1.ANT, 2.MED). These concepts are abstract, supported only statistically and contain little biological information. Moreover, selection operates on actual populations; it is populations that evolve, but not individual organisms, phylogenetic branches or evolutionary lines. Therefore, when reconstructing the history of a ‘young’ plague microbe unique to the *Enterobacteriaceae* family, which sharply deviated from the main course of evolution of intestinal inhabitants, the principles of population way of thinking and adaptatiogenesis should not be ignored.

Admittedly, each of the considered approaches has its pros and cons, while the resolution of existing contradictions is the sole way to find the truth. Further integration of molecular, genetic and ECO approaches will not only contribute to the investigation of the history of plagues but also lay the groundwork for advancement of the theory of molecular evolution of the plague pathogen and many other pathogenic microorganisms.

## Use of AI tools declaration

The authors declare they have not used Artificial Intelligence (AI) tools in the creation of this article.
